# *Tbx4* and *Tbx5* acting in connective tissue are required for limb muscle and tendon patterning

**DOI:** 10.1016/j.devcel.2009.11.013

**Published:** 2010-01-19

**Authors:** Peleg Hasson, April DeLaurier, Michael Bennett, Elena Grigorieva, L. A. Naiche, Virginia E. Papaioannou, Timothy J. Mohun, Malcolm P.O. Logan

**Affiliations:** 1 Division of Developmental Biology, MRC-National Institute for Medical Research, Mill Hill, London NW7 1AA, UK.; 3 Division of Developmental Neurobiology, MRC-National Institute for Medical Research, Mill Hill, London NW7 1AA, UK.; 4 Columbia University, College of Physicians and Surgeons, Department of Genetics and Development, 701 W. 168th St., New York, NY 10032, USA.

## Abstract

Proper functioning of the musculo-skeletal system requires the precise integration of bones, muscles and tendons. Complex morphogenetic events ensure that these elements are linked together in the appropriate 3D configuration. It has been difficult, however, to tease apart the mechanisms that regulate tissue morphogenesis. We find that deletion of *Tbx5* in forelimb (or *Tbx4* in hindlimbs) specifically affects muscle and tendon patterning without disrupting skeletal development thus suggesting that distinct cues regulate these processes. We identify muscle connective tissue as the site of action of these transcription factors and show that N-Cadherin and β-Catenin are key downstream effectors acting in muscle connective tissue regulating soft-tissue morphogenesis. In humans, *TBX5* mutations lead to Holt-Oram syndrome, which is characterised by forelimb musculo-skeletal defects. Our results suggest that a focus on connective tissue is required to understand the aetiology of diseases affecting soft tissue formation.

Dissecting the cues involved in patterning specific tissues in the developing embryo has proven to be a challenge. The vertebrate limb has been a useful model to study these processes and much effort has been aimed at identifying the cues that pattern the limb skeleton (Mariani and Martin, 2003). For the limb skeleton to function properly it is critical that the appropriate associated muscles become anchored to the skeletal scaffold via the correct tendons. These three tissues must interact with each other in 3-dimensional space with high fidelity to form a functional musculoskeletal system. Although much is known about the molecular pathways that determine muscle cell fate and subsequent differentiation (Biressi et al., 2007), very little is known about the mechanisms that regulate morphogenesis of individual muscles and their associated tendons.

The T-box transcription factors *Tbx5* and *Tbx4* are expressed in the forelimb- and hindlimb-forming regions, respectively, of the lateral plate mesoderm prior to and during limb bud initiation. Once limb buds have formed, both genes are expressed broadly in the limb mesenchyme (Logan et al., 1998; Rallis et al., 2003) until later stages of development (Hasson et al., 2007; Naiche and Papaioannou, 2007b). Their temporal and spatial expression pattern suggests that *Tbx5* and *Tbx4* may have roles in limb patterning processes. Mutations in human, *TBX5* and *TBX4*, are associated with Holt-Oram syndrome (HOS) (Basson et al., 1997; Li et al., 1997), and Small Patella syndrome (SPS) (Bongers et al., 2004), respectively, and both syndromes are characterised by various limb defects in addition to other abnormalities.

Using a conditional deletion strategy in mice and 3D imaging techniques (Sharpe et al., 2002; Weninger and Mohun, 2002), we show that in the absence of *Tbx5* in the forelimb and *Tbx4* in the hindlimb, limb muscle and tendon patterning is disrupted. Although limb muscles undergo terminal differentiation and myotubes fuse to form muscle bundles, the muscles that form in these mutant limbs are the incorrect size and shape, undergo abnormal splitting and insert at the inappropriate locations on bone. The associated tendons also show abnormal patterning. Significantly, the limb skeleton is not affected by this *Tbx5/Tbx4*-deletion regime (Hasson et al., 2007; Naiche and Papaioannou, 2007b) indicating that the patterning of these tissues can be separated from one another and suggesting that cues required for muscle and tendon patterning are independent from those of the skeleton. This is consistent with the observation that some muscle phenotypes in HOS patients are not associated with corresponding skeletal abnormalities (Newbury-Ecob, ; Newbury-Ecob et al., 1996). We identify the temporal developmental window in which this activity is carried out and demonstrate that reduction of N-Cadherin and β-Catenin causes muscle connective tissue deformities that account for this phenotype. This study reveals a molecular mechanism affecting soft tissue patterning in the limb shedding light on the previously unappreciated role of connective tissues in development of and diseases affecting the limb musculoskeletal system.

## Results

### *Tbx5* and *Tbx4* are required for limb muscle patterning

*Tbx5* is expressed in the cells of the lateral plate mesoderm that ultimately form the forelimb bud and is known to be an essential component of the genetic cascade that triggers limb initiation since in its absence no forelimb bud forms (Agarwal et al., 2003; Ahn et al., 2002; Rallis et al., 2003). *Tbx4* appears to have an equivalent role in the hindlimb (Minguillon et al., 2005) and, although a nascent bud does form in the *Tbx4* homozygous null mutant, it fails to develop further (Naiche and Papaioannou, 2003). Both *Tbx5* and *Tbx4* continue to be expressed broadly in the limb bud mesenchyme beyond limb initiation stages, however neither gene is required to maintain outgrowth and patterning of the limb skeleton. To test whether *Tbx5* and *Tbx4* are required for patterning other tissues of the musculoskeletal system, namely the muscles and tendons, we carried out conditional deletion of these genes at early stages of limb outgrowth (E8.5-E12.5) and subsequently monitored muscle and tendon development.

**Figure 1:**
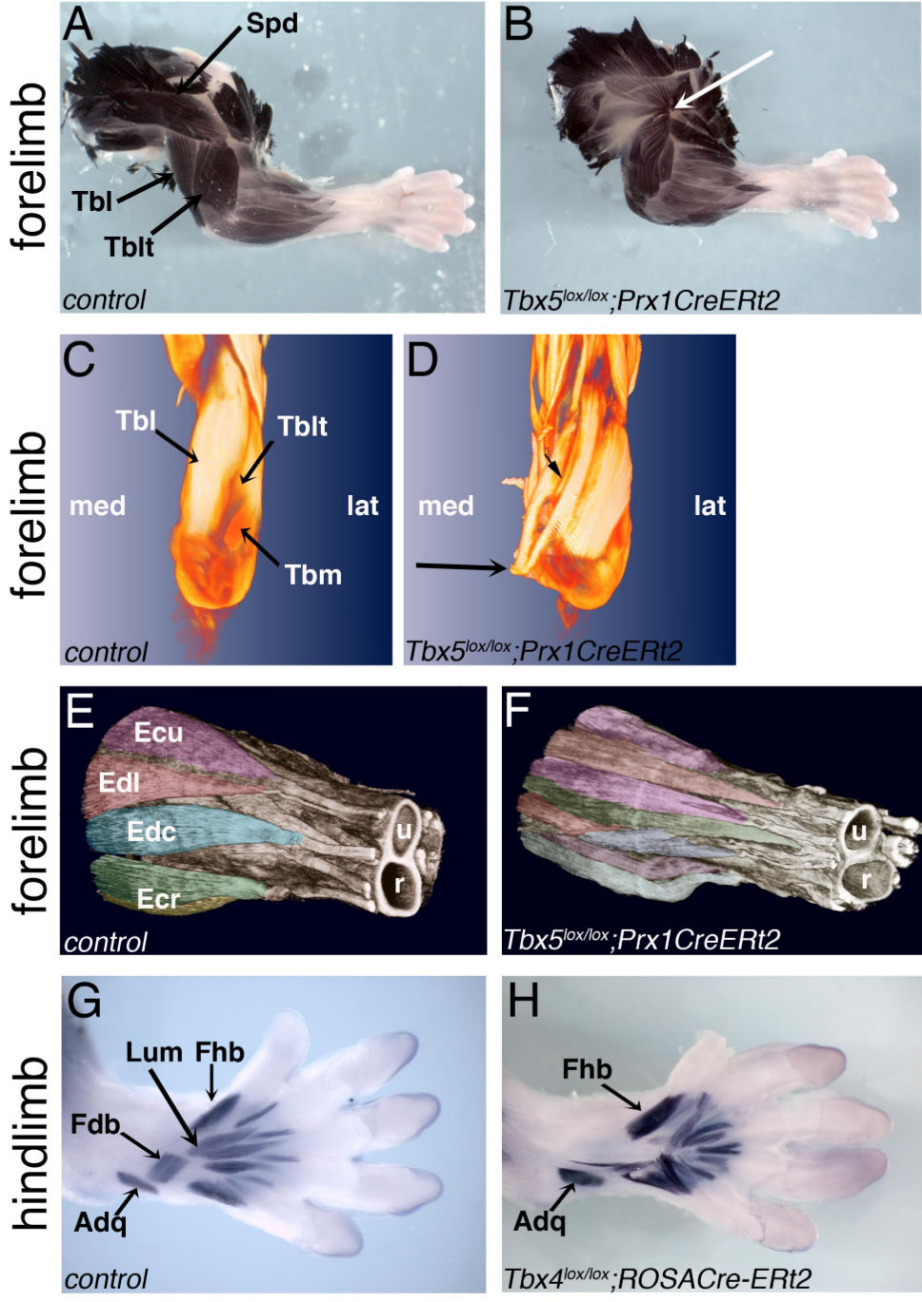
Limb muscle pattern is disrupted following deletion of Tbx5 or Tbx4 Whole mount immunohistochemistry of E16.5 forelimbs (A-D) or E15.5 hindlimbs (G,H) with the anti-muscle myosin antibody. The normal pattern of forelimb muscles (A) is disrupted in the *Tbx5* mutant (*Tbx5^lox/lox^;Prx1CreERt2*) (B) (dorsal views). Still images of an OPT, 3D analysis, viewed from the back of the upper arm, comparing control (C) and *Tbx5*-mutant (D) forelimbs showing ectopic splitting (small arrow) and insertions of muscles (long arrow) at the region of the Tbl, Tblt and Tbm. Dorsal view using HREM of control (E) and *Tbx5*-mutant forelimb showing ectopic splitting of muscles in the region of the zeugopod (F) (individual muscle bundles have been shaded for clarity, however colours do not indicate muscle type). Muscle pattern in the ventral footplate (G) is disrupted in the *Tbx4*-deleted hindlimb (H). Control littermates shown are *Tbx5^lox/+^;Prx1CreERt2* (A,C) or *wild-type* (E,G). CreERt2 was activated by TM administration at E10.5 (A,B,G,H) or at E9.5 (C-F). Spd=M. spinodeltoideus, tbl=M. triceps brachii (long), Tblt=M. triceps brachii (lateral); Tbm=M. triceps brachii (medial). Amg=M. Adductor magnus, Sm=M. semimembranosus, Gra=M. gracilis anticus, Ecu=M. extensor carpi ulnaris, Edl=M. extensor digitorum lateralis, Edc=M. extensor digitorum communis, Ecr=M. extensor carpi radialis (longus and brevis), r= radius, u= ulna, Lum=M. lumbricales, Adq=M. abductor quinti, Fhb=M. flexor hallucis brevis, Fdb=M. Flexor digitorum brevis. See also Table S1, Movies S1 and S2 and Figure S1.

By E14.5, tissues of the musculoskeletal system have largely assumed their mature arrangements and individual muscle bundles and associated tendons are identifiable (DeLaurier et al., 2006). To follow muscle pattern, we used an antibody that enables us to identify terminally differentiated muscles using whole mount staining (Fig.1). Deletion of *Tbx5* and *Tbx4* between E9.5 and E10.5 leads to equivalent disruptions of normal muscle splitting patterns and muscle sizes and alters the sites of individual muscle origins and insertions. For example in the wild-type forelimb, the spinodeltoidus (Spd), the M. Triceps brachii longus (Tbl) and the M. Triceps brachii lateralis (Tblt) have characteristic origin and insertion sites (Fig. 1A). In the *Tbx5* mutant, the muscle bundles in the equivalent region have a common origin at one focus (Fig. 1B, white arrow) and have split to form smaller muscle bundles that insert at aberrant positions. Significantly, if deletion regimes are carried out at later stages (E11.5 for *Tbx5*, E12.5 for *Tbx4*; Table S1) limb development is apparently unaffected. To analyse the muscle splitting and insertion phenotypes in more detail we carried out a 3D analysis of muscle morphology using Optical Projection Tomography (OPT) (DeLaurier et al., 2006; Sharpe et al., 2002) and High Resolution Episcopic Microscopy (HREM) (Weninger and Mohun, 2002). As an example of abnormal muscle splitting and insertion in the mutant, we focused on the three triceps muscles of the forelimb, Tbl, M. triceps brachii medialis (Tbm) and Tblt, that insert on the olecranon process of the ulna (Fig. 1C). In the *Tbx5* mutant, the muscles in the equivalent region have split into additional bundles (short arrow), some of which now insert in the more distal shaft of the ulna (Fig. 1D, long arrow and movies S1,S2). Zeugopodal muscles, such as those occupying the region of the M. extensor digitorum communis (Edc), are similarly ectopically split in the *Tbx5*-deleted limb (Fig. 1E,F). This perturbation of normal muscle pattern can also be observed in the muscles of the autopods (not shown). Similar muscle mispatterning is also observed in *Tbx4* mutant hindlimbs. For example, in the analogous region to the M. Lumbricales (Lum) and M. flexor digitorum brevis (Fdb) of the wild-type hindlimb (Fig. 1G) muscles in the *Tbx4* mutant mis-insert and assume the wrong shape (Fig. 1H).

Following conditional deletion of *Tbx5* and *Tbx4*, all of the limb musculature is affected and it is not possible to detect any limb muscles that retain all aspects of their normal pattern. Terminal differentiation of the limb muscle, however, is not apparently affected in either *Tbx5* or *Tbx4* mutants. The sarcomeric marker, muscle myosin is expressed normally as judged by immunohistochemical staining (Fig. 1), myoblasts undergo fusion to form muscle fibres and bundles and the cytoarchitecture of the sarcomere in the *Tbx5* mutant limbs, analysed by transmission electron microscope, is not affected (not shown). Significantly, although deletion of *Tbx5* from E10.5 leads to dramatic muscle and tendon patterning defects (see below), formation and patterning of the limb skeleton is unaffected (Hasson et al., 2007) (not shown, Table S1). Likewise, conditional deletion of *Tbx4* at E10.5 produces some minor skeletal abnormalities but deletion at E11.5 leads to muscle mispatterning without affecting the skeleton (Naiche and Papaioannou, 2007b) (Table S1) indicating that at least some aspects of the patterning of two elements of the musculoskeletal system, the muscles and tendons (see below), can be uncoupled from that of the skeleton. Comparable muscle phenotypes are obtained when a dominant-negative form of *Tbx5* (*Tbx5*-EN) (Rallis et al., 2003) is misexpressed in chick wings (Fig. S1) suggesting that the underlying molecular mechanisms regulated by *Tbx5* (and by inference *Tbx4*) in limb muscle patterning are conserved across vertebrates.

### *Tbx5* regulates tendon patterning

**Figure 2:**
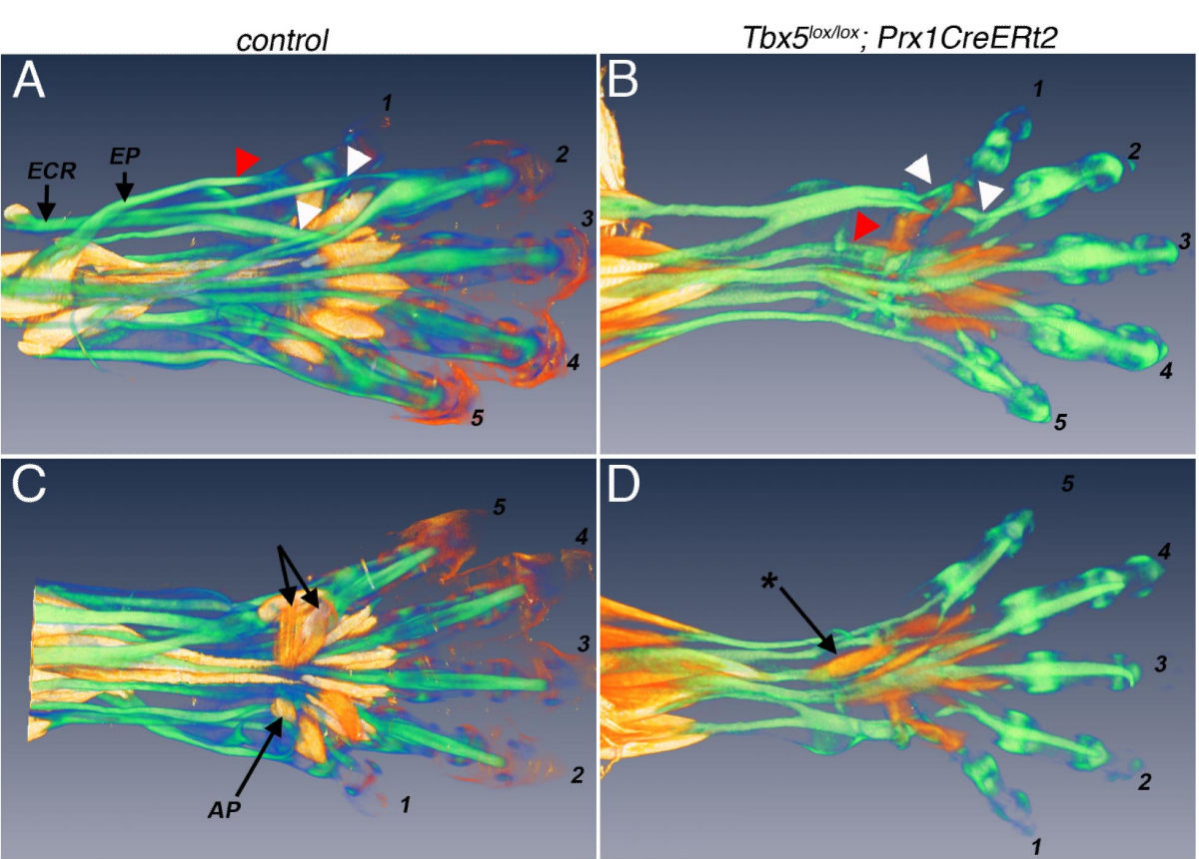
Mispatterning of tendons does not prevent their interactions with muscles and skeleton OPT analysis of whole mount immunohistochemistry of E15.5 control (A,C) and *Tbx5*-deleted autopods (B,D) of which TM was administered at E9.5 showing mispatterning of muscles (red) and tendons (green). Control littermates shown are *Scx-GFP* (A,C). A,B dorsal views; C,D ventral views. Some tendons and muscles are designated. Note the lack of analogous muscles and tendons in the mutant limbs. ECR=M. extensor carpi radialis, EP=M. extensor pollicis, AP=M. Abductor pollicis. *= ectopic muscle. Triangles mark tendons that in control limbs insert onto digit 1 (red) and digits 2 and 3 (white) but mis-insert in the mutant.

The limb muscles are connected to the limb skeleton via tendons. For the correct musculoskeletal pattern to be elaborated, specific groups of tendon progenitors must associate with the appropriate muscle bundles before they make their attachment to the skeleton. Experiments in the chick to generate either muscle-less or limbs lacking specific tendons demonstrated that patterning of each tissue is initially independent, but at later stages the two tissues become inter-dependent (Kardon, 1998). To follow muscle origins and insertions and hence the interactions of muscles with tendons, we deleted *Tbx5* by tamoxifen (TM) administration at E9.5 in mice that also carry a *Scx-GFP* transgene that marks tendons and their progenitors (*Tbx5^lox/lox^;Prx1CreERt2;Scx-GFP*) and analysed both muscle and tendon pattern in 3D using OPT (Sharpe et al., 2002) (Fig. 2). Deleting *Tbx5* at this stage gives rise to limbs with minor skeletal deformities similar to those commonly seen in HOS individuals, such as triphalangeal thumb. Muscle pattern is altered similarly to that shown in Fig.1 and, in addition, disruption of tendon pattern is also visible (Fig. 2). The normal pattern of tendon fibres that connect forearm (zeugopodal) muscles to the skeletal elements of the handplate (Fig. 2A,C) is unrecognisable in the *Tbx5* mutant (Fig. 2B,D). Fewer tendon fibres are present; some are thinner than normal, while some have fused. Significantly, mispatterned muscles make myotendinous attachments to tendons and the tendons develop entheses on the forming skeleton, indicating that the signals required for the crosstalk between muscle and tendon and tendon and bone, enabling these fundamental interactions, remain functional in the mutant.

### Early alterations of muscle and tendon pattern

The majority of the myoblasts have migrated into the forelimb by E10.5 (33 somites) (Houzelstein et al., 1999). Previously, we have shown that following TM administration, 18-24 hours are required for full Cre activity from the *Prx1CreERt2* transgene (Hasson et al., 2007). Deletion of *Tbx5* during or after myoblasts have migrated into the limb (i.e. TM administration at E9.5 and E10.5, respectively) results in similar muscle patterning defects suggesting that the muscle phenotypes are not the result of the myoblasts failing to migrate properly. Consistent with this interpretation, we find that even in limbs in which TM is administered at E8.5 to delete *Tbx5* in the limb at stages when myoblast progenitors first migrate into the limb, *Pax3* expression in the limb is unaffected at E10.5 (not shown). Furthermore, the *Tbx5*- and *Tbx4*-deleted limbs do not show a reduction in muscle mass in comparison to control littermates (e.g. Fig. 1) or in *MyoD* staining at E12.5 (see below) therefore the total number of myoblasts migrating into the limb in both mutants and wild-type appear to be the same. Taken together, these results suggest that *Tbx5* and *Tbx4* do not regulate initial migration of myoblast progenitors into the limb.

**Figure 3:**
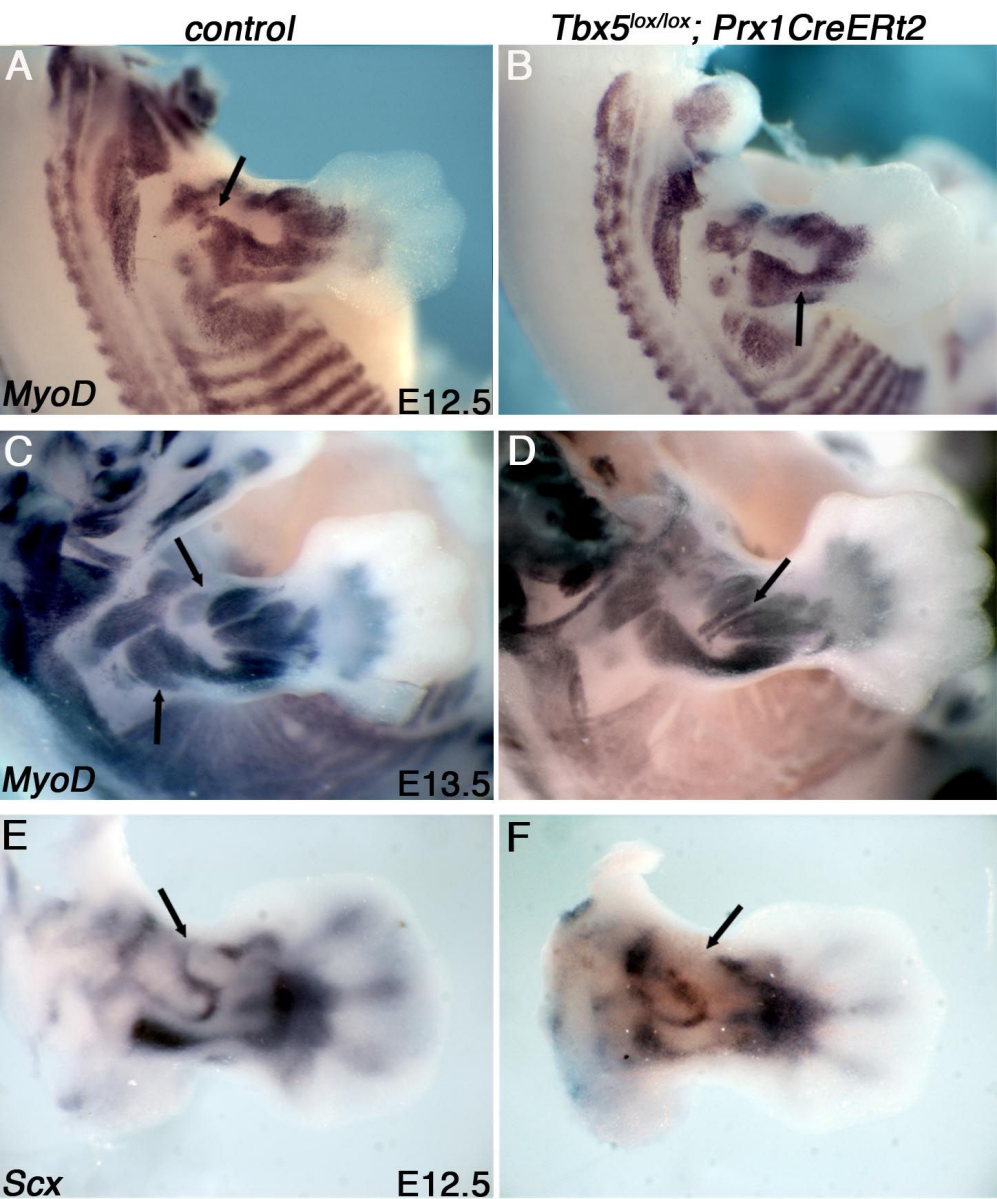
Tbx5 exerts its muscle and tendon patterning activity around E11.5-E12.5 Whole mount in situ hybridization for *MyoD* at E12.5 reveals subtle differences between control(Aand *Tbx5*-deleted limbs (B). Aberrant splitting of nascent muscle bundles (arrowed in D) is clear at E13.5(C,D). Similarly, tendon progenitors, monitored by *Scx* expression, are mispatterned in the *Tbx5*-mutant limbs by E12.5 (E,F). Control littermates shown are *Tbx5^lox/+^;Prx1CreERt2* (A,C,E). TM was administered to pregnant females at E10.5 and embryos harvested at E12.5-E13.5. See also Figure S2.

To identify the underlying mechanism by which *Tbx5* and *Tbx4* exert their muscle- and tendon-patterning activity we first wished to identify the temporal window of their activity and the earliest observable defects following their deletion. After TM administration into pregnant females at E10.5 we harvested litters at E12.5 and stained for muscle (*MyoD*) and tendon-markers (*Scx*). As early as E12.5, when muscle splitting and subdivision into distinct muscle bundles can first be observed, the expression patterns of both *MyoD* and *Scx* are abnormal in *Tbx5* mutants (Fig. 3A,B,E,F; arrows) and *Tbx4* mutants (Fig. S2). These phenotypes are further enhanced by E13.5 when the pattern of the emerging muscles is altered from wild-type and ectopic splitting of nascent muscle bundles is observed (Fig. 3C,D; note arrow, Fig. S2). These results indicate that the muscle and tendon phenotypes observed at E15.5-E16.5 are caused by a disruption of earlier *Tbx4/Tbx5*-dependent processes that occur at around E11.5-E12.5, but not earlier. Significantly, these results demonstrate that *Tbx5* and *Tbx4* regulate muscle and tendon patterning before E12.5, when the progenitor pools of these two tissues are developing independently of each other (Kardon 1998).

### *Tbx5* regulates muscle patterning in a non-autonomous manner

Our results demonstrate that *Tbx5* and *Tbx4* have roles in coordinating forelimb and hindlimb muscle pattern, respectively. To further understand the activity of these genes, we wished to identify the cells in which they are acting. Classical embryology and recent molecular data suggests that extrinsic cues are required for patterning limb muscles (e.g. (Christ et al., 1977; Kardon et al., 2003) although there is some data suggesting some non-limb myoblasts are patterned by intrinsic cues (Alvares et al., 2003). The *Prx1Cre* deleter line is expressed in all limb mesenchymal cells including the myoblast progenitors once they migrate into the limb (Durland et al., 2008; Logan et al., 2002) and therefore cannot distinguish between autonomous and non-autonomous Tbx5 activity. To overcome this problem, we took advantage of the *Pax3CreKI* deleter line in which *Cre* is inserted into the *Pax3* locus (Engleka et al., 2005) that enables Cre activity, and hence *Tbx5* deletion, in the myoblasts prior to their migration into the limb field, rendering all limb myoblasts *Tbx5* null. Deletion of *Tbx5* in the myoblasts does not affect their patterning (Fig. S3), demonstrating that *Tbx5* controls limb muscle-patterning non-autonomously, consistent with the model that extrinsic cues are critical for muscle morphogenesis.

### *Tbx5* regulates muscle connective tissue organisation

**Figure 4:**
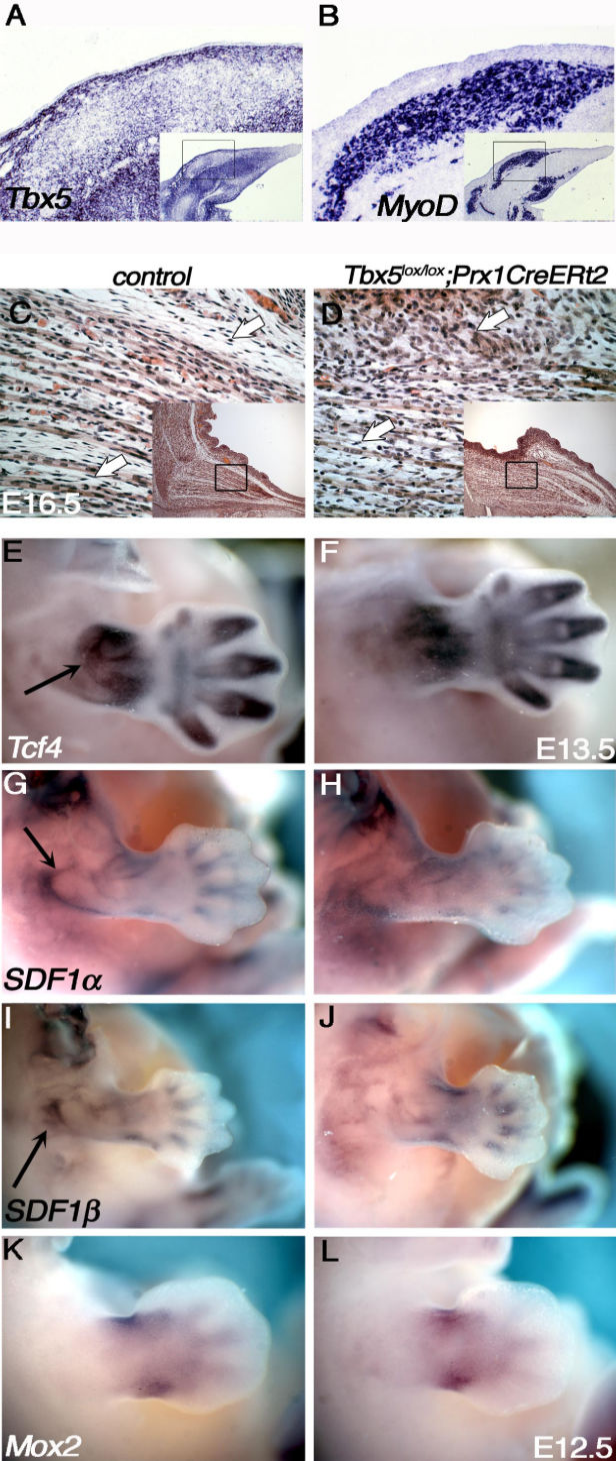
Candidate muscle patterning genes are still expressed following deletion of Tbx5 In situ hybridisation of serial sections at E12.5 of *Tbx5* (A) and *MyoD* (B)*Tbx5* is expressed in the cells ensheathing and embedded within the muscles. H&E staining comparing the histology of control (C) and Tbx5 mutant (D) limbs.The connective tissue (white arrows) is disorganized in the mutant. The black box within the smaller inset indicates the region covered by the magnified view. No gross changes in expression levels of *Tcf4* (E,F), *SDF1α* (G,H) or *SDF1β* (I,J) by E13.5 or *Mox2* (K,L) (by E12.5) are detected by whole mount in situ between control and mutant limbs. Black arrows point to normal domains of expression in the control (E,G,I) that are altered in the mutant examples (F,H,J). TM administration at E10.5 (panels C,F,H,J) or E9.5 (panel L). See also Figure S3.

The results above demonstrate that *Tbx5* does not function autonomously to pattern the limb muscles. A possible alternative explanation is that *Tbx5* acts in muscle connective tissue (MCT), found adjacent to forming muscles and which has been shown to influence muscle formation (Grim and Wachtler, 1991). *Tbx5* is strongly expressed in MCT cells that are embedded within and ensheath the *MyoD*-positive muscle progenitors (Fig. 4A,B). To test the function of *Tbx5* in MCT we deleted this gene and analysed the MCT at E16.5, a stage when it can be identified histologically. Deletion of *Tbx5* leads to a disruption of normal MCT organisation (Fig. 4C,D; arrows).

We also analysed the expression patterns of a battery of molecular markers that have been implicated in limb MCT development or muscle patterning following deletion of *Tbx5*. Recently, Tcf4, a nuclear component of the canonical Wnt signalling pathway, has been shown to be expressed in MCT and tendon progenitors and to be involved in determining the basic pattern of limb muscles (Kardon et al., 2003). *Tcf4* expression is still detectable in the *Tbx5* mutant limbs demonstrating that MCT is still present, however the distribution of *Tcf4*-positive cells is altered (Fig. 4E,F). We also analysed other genes expressed in limb mesenchyme or MCT that have been implicated in limb muscle formation, such as *SDF1α* and *SDF1β* (Vasyutina et al., 2005), *Mox2* (Mankoo et al., 1999), *SF/HGF* (Dietrich et al., 1999), *Lbx1* (Schafer and Braun, 1999), *Osr1* and *Osr2* (Stricker et al., 2006), *BMP2/4* (Bonafede et al., 2006) and they all showed the same features in that they continued to be expressed in the mutant although their pattern of expression was altered (Fig. 4G-L and not shown).

**Figure 5:**
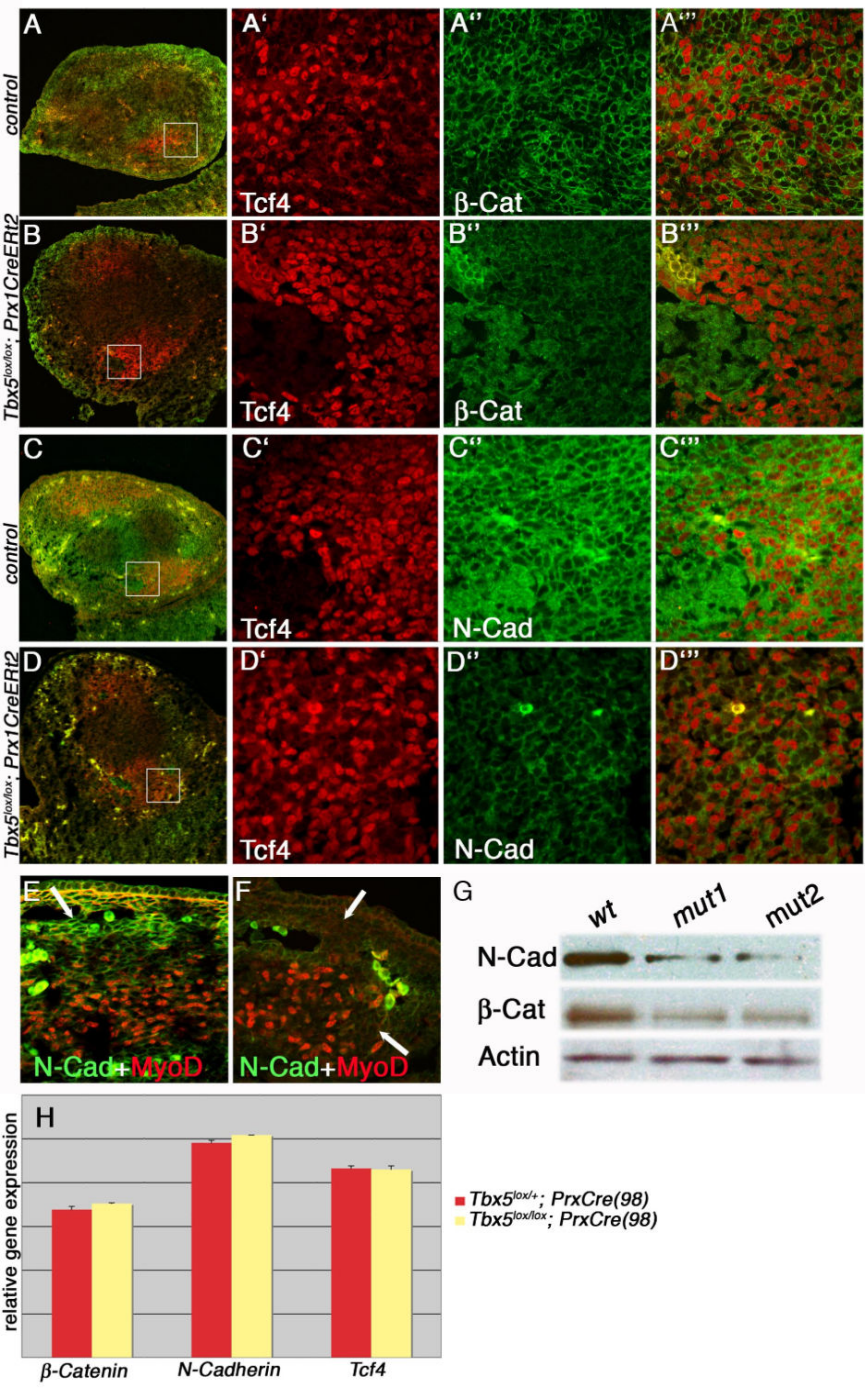
*Tbx5 regulates* β-Catenin *and* N-Cadherin *in muscle connective tissue* Immunohistochemistry of transverse sections of limbs at E12.5. β-Catenin (green) is localised at the cell membrane of Tcf4 (red)-positive MCT cells (A). Membrane localized β-Catenin (green) is lost in Tbx5 mutant Tcf4 (red)-positive MCT cells (B. N-Cadherin (green) expression is localised at the cell membrane of Tcf4-expressing MCT cells (Red) (C). Membrane localized N–Cadherin (green) is lost in Tbx5 mutant Tcf4 (red)-positive MCT cells Tagged panels show high magnifications of boxed areas (A-D). Saggital sections of control (E) and *Tbx5*-deleted (F) limbs stained with MyoD (red) to mark the muscles. N-Cadherin (green) expression in the cells ensheathing the muscles in control (E, white arrow) is not present in the mutant (F, white arrows)(E,F; arrows; bright green cells are autofluorescent red blood cells). Western blot analysis from *wild-type* or two different*Tbx5* mutant limbs confirms the reduction of N-Cadherin and β-Catenin (G). qPCR analysis showing *β-Catenin*, *N-Cadherin* and *Tcf4* transcripts levels found in control (red bar) do not changes in the Tbx5 mutant (yellow bar) (H). Error bars mark standard deviation. See also Figure S4

In *Tbx5* conditional mutants, MCT is disorganised throughout the limb (e.g. Fig. 4D), consistent with our observations that all limb muscles and tendons appear to be affected. This suggests that *Tbx4*/*Tbx5* regulate a fundamental process within the limb mesenchyme, such as cell:cell adhesion, a process known to play key roles in development and tissue morphogenesis (Gumbiner, 2005). Several classes of proteins and signalling cascades have been shown to participate in cell adhesion. Among these, β-Catenin is a focal player having major roles in both cell:cell adhesion as well as signalling (Ben-Ze’ev and Geiger, 1998; Brembeck et al., 2006). Furthemore, Wnt signalling has been implicated in limb muscle patterning in the MCT via the activity of Tcf4 (Kardon et al., 2003) as well as in limb muscle development (Anakwe et al., 2002). In *wild-type* E12.5 limbs, β-Catenin is clearly detectable at the cell membrane in the Tcf4-expressing MCT cells, whereas, in *Tbx5*- and *Tbx4*-deficient limbs there is a marked decrease in its levels at the cell membrane (Fig. 5A,B and Fig. S4). No difference in *β-Catenin* transcription is observedbetween control and *Tbx5*-deleted limbs using in situ hybridisation (not shown). To verify and quantify these observations, we performed quantitative PCR. No difference is observed in *β-Catenin* transcript levels in *Tbx5*-deleted limbs (see Experimental Procedures) and their heterozygous littermate controls (Fig. 5H) suggesting that the disruption of β-Catenin expression in *Tbx4*- and *Tbx5*-deleted limbs is not at the transcriptional level.

Following the loss of membrane-tethered β-Catenin, a concomitant reduction of its membranal anchors, the Cadherins, are observed (Cali et al., 2007). Like β-Catenin, Cadherins participate in multiple processes and play cardinal roles in cell adhesion (Halbleib and Nelson, 2006). Consequently, we tested whether certain Cadherins are also affected in the *Tbx4*- and *Tbx5*-deleted limbs. In E12.5 limbs in which *Tbx5* has been deleted at E8.5 there is a marked reduction in pan-Cadherin antibody staining (not shown) suggesting that one or more Cadherins are affected. N-cadherin, is a classical, mesenchymally-expressed Cadherin, which has been suggested to participate in limb myoblast pathfinding (Brand-Saberi et al., 1996). Following deletion of *Tbx5* or *Tbx4*, N-Cadherin is down-regulated in Tcf4-positive MCT cells (Fig. 5C,D). RNA in situ staining and qPCR analysis confirmed that as with β-Catenin, *Tbx4/5* regulation of N-Cadherin is not at the transcriptional level (not shown and Fig. 5H). Finally, *Tcf4* transcription was previously shown to be downstream of Wnt signalling (Kardon et al., 2003), however, neither RNA in situ hybridisation (Fig. 4E,F) nor qPCR showed any difference in its levels following the deletion of *Tbx5* (Fig. 5H), reinforcing a model that reduction of β-Catenin in the MCT does not affect Wnt signalling.

To further characterise this reduction, we marked the forming muscles with MyoD and co-stained with N-Cadherin. As expected, a strong reduction is observed in the cells ensheathing the muscles (Fig. 5E,F). Western blots from *wild-type* and *Tbx5* mutant limbs using N-Cadherin and β-Catenin antibodies confirmed a decrease in the levels of both proteins in the mutant (Fig. 5G). Expression levels of another mesenchymally-expressed Cadherin, Cadherin 11, are unaffected however (not shown) suggesting that there is not a general Cadherin downregulation and that the response to the loss of β-Catenin in MCT may be limited to N-Cadherin.

**Figure 6:**
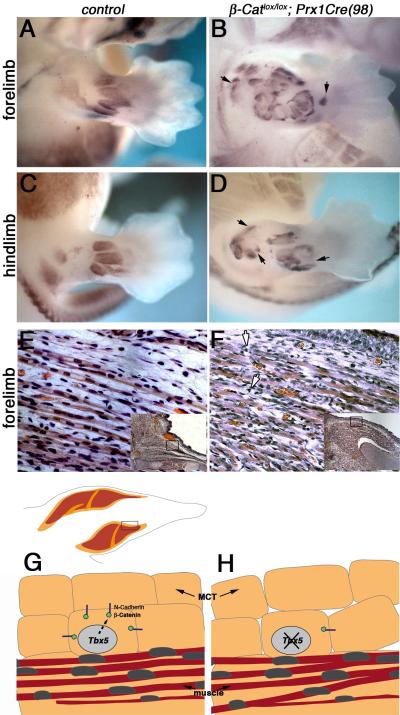
Disrupting the β-Catenin/N-Cadherin complex leads to MCT disorganization and to ectopic muscle splitting and mispatterning Whole mount in situ hybridization for *MyoD* on E13.5 limbs to detect the forming muscles in control (*β-Catenin^lox/+^;Prx1Cre(98)* A,C) compared to mutant (*β-Catenin^lox/lox^;Prx1Cre(98)* B,D) in forelimbs and hindlimbs. Histological (H&E) staining of an E16.5 control (*wild-type*) (E) and *β-Catenin^lox/lox^;Prx1Cre(98)* forelimb shows that the MCT (arrowed) is disorganized (note white arrows) (F). Model depicting activity of *Tbx4/Tbx5* in normal MCT (orange) and underlying muscles (red) in the developing limb (G). In *wild-type* limbs, *Tbx5* (and *Tbx4*) expressed in the MCT facilitate the expression of N-Cadherin and β-Catenin required for MCT organisation and integrity. In the *Tbx5* (and *Tbx4*) mutant limbs, a strong reduction in N-Cadherin and β-Catenin is observed leading to the disorganization of the MCT and mispatterning of the adjacent muscles (H).

Recently it has been reported that β-Catenin is not required cell-autonomously within limb muscles for their embryonic development and patterning (Hutcheson et al., 2009). Our results suggest N-Cadherin/β-Catenin expressed in MCT have a role in muscle patterning. To directly test this model, we used a *β-Catenin* conditional allele in combination with a cre transgenic (*Prx1Cre(98)*) to delete β-Catenin activity in the limb bud mesenchyme. Embryos were harvested at E13.5 and the limb buds analysed for *MyoD* expression to assess whether the forming muscles were mispatterned. Deletion of *β-Catenin* in the limb mesenchyme leads to ectopic muscle splitting and muscle mispatterning in both forelimbs and hindlimbs (Fig. 6A-D) presumably due to the disruption of MCT organisation, similar to that observed in the *Tbx5* mutant limbs (Fig. 6E,F, cf. with Fig. 4D) and consistent with a model in which the N-Cadherin/β-Catenin complex in the MCT is critical for muscle patterning.

## DISCUSSION

Our results reveal a spatio-temporal window in which *Tbx4* and *Tbx5* are required for patterning the soft tissues (muscles and tendons) of the musculoskeletal system. *Tbx4* and *Tbx5* exclusively regulate muscle and tendon patterning while having no apparent effect on the generation, proliferation or migration of the progenitors of these tissues, strongly suggesting that they regulate a distinct patterning signal(s), which our results indicate are dependent on proper organisation of MCT. Regulation of the Tcf4-expressing connective tissue can account for the independent patterning activity Tbx5 and Tbx4 have on both muscles and tendons since Tcf4 is also expressed in domains where tendon progenitors arise (Kardon et al., 2003). We propose a model in which *Tbx4/Tbx5* expressed in the MCT positively regulate expression of N-Cadherin and β-Catenin that are required for the proper integrity and organisation of this tissue that in turn is critical for correct patterning of the adjacent muscles (Fig. 6F) and tendons. The loss of *Tbx4/Tbx5*, leads to a downregulation of N-Cadherin and β-Catenin, disorganisation of the MCT, resulting in mispatterning of muscles (Fig. 6G). Consistent with this model, we show that deletion of β-Catenin results in similar phenotypes.

N-Cadherin has been previously implicated in limb myoblast pathfinding (Brand-Saberi et al., 1996). In addition, Cadherins and β-Catenin expressed in craniofacial connective tissue have been suggested to play a role in patterning adjacent head muscles (Rinon et al., 2007). Interestingly, *β-Catenin^lox/lox^; Prx1Cre(98)* mice also exhibit some cranial muscle mispatterning (not shown) since this Cre-deleter is also expressed in the ventral part of the 1^st^ branchial arch (Logan et al., 2002). Together these data suggest that Cadherin/β-Catenin activity in connective tissue could be a general mechanism regulating vertebrate muscle patterning. Recent data suggests that like β-Catenin, N-Cadherin does not function solely in cell adhesion, but has many other roles, such as in cell signalling and transcriptional regulation (Halbleib and Nelson, 2006). A major challenge now will be to precisely determine how the N-Cadherin/β-Catenin complex functions in connective tissue to regulate muscle and tendon morphogenesis.

*Tcf4* is expressed in muscle connective tissue but not myoblasts themselves and has also been implicated in muscle patterning and formation. *Tcf4* continues to be expressed in *Tbx5* mutants suggesting it either acts in a parallel pathway or potentially upstream. Although the *Tbx5*-dependent β-Catenin reduction we observe could, in principle, effect Tcf4/Wnt signalling in the MCT, all our data suggests this is not the case. First, following the deletion of β-Catenin protein levels of its associated membranal cofactor N-Cadherin are reduced in a Wnt-independent manner (Cali et al., 2007). Second, blocking the Wnt pathway using dominant negative Tcf4 (Tcf4-EN) affects myotube differentiation, leading to some muscles failing to form while the patterning of other muscle is affected variably (Kardon et al., 2003). In contrast, in the *Tbx4*/*Tbx5*-deleted limbs all muscles are uniformly affected and there is no effect on muscle differentiation. Finally, *Tcf4* mRNA was shown to be responsive to Wnt signalling (Kardon et al., 2003) however, we do not detect any changes in *Tcf4* transcript levels following the deletion of *Tbx5* (Fig. 5H). Collectively, these differences suggest that the *Tbx4/Tbx5*-dependent β-Catenin loss in the MCT affects muscle patterning in a mechanism that is distinct from the Wnt-dependent Tcf4 pathway.

*Tbx4* and *Tbx5* have equivalent roles in initiating limb outgrowth during a narrow, early time-window at around E9.0 (Hasson et al., 2007; Naiche and Papaioannou, 2007b) Our current results demonstrate that at later stages of limb development when the genes are no longer required to initiate limb outgrowth, both genes have a role patterning the limb muscles and tendons. This second pulse of activity lasts for 24-48 hours. Limb muscles are formed from a subpopulation of the hypaxial myoblasts that migrate into the limb buds and it is once they have entered this environment that these cells receive instructive cues that dictate ultimate muscle morphology (Buckingham et al., 2003). Our data suggests that *Tbx4* and *Tbx5* have been co-opted to pattern limb muscles by regulating a general Cadherin/β-Catenin-dependent muscle patterning “cassette” after myoblast migration has terminated and co-incident with the onset of terminal differentiation. Little is known about the tissue interactions that occur during tendon patterning and the deletion approaches we have taken do not distinguish whether these *T-box* genes are acting autonomously or non-autonomously on tendon progenitors.

Together, our results point to MCT organisation and integrity being critical for normal patterning of soft tissues. Accordingly, we suggest that disruption of MCT development, and specifically the Cadherin/β-Catenin complex, play a role in human soft tissue pathologies. In humans, HOS patients can present with soft tissue abnormalities that are not associated with skeletal defects (Newbury-Ecob, ; Newbury-Ecob et al., 1996; Spranger et al., 1997) consistent with the observations that, despite the widespread soft tissue defects produced in our mouse models following deletion of *Tbx5/4*, the skeleton could be unaffected. We propose that defects in muscle connective tissue integrity should be explored as an explanation for soft tissue abnormalities and the influence of connective tissue considered in developing strategies for musculoskeletal tissue regeneration therapies.

## Experimental Procedures

### Transgenic mice and embryos

Mouse embryos were staged according to Kaufman (Kaufman, 2001). Noon on the day a vaginal plug was observed was taken to be embryonic day (E) 0.5. The mouse lines carrying a conditional allele of *Tbx5* (Bruneau et al., 2001), of *Tbx4* (Naiche and Papaioannou, 2007b), *β-Catenin* (Huelsken et al., 2001), *Scx-GFP* (DeLaurier et al., 2006), *RosaCreERt2* (de Luca et al., 2005) and a *Prx1CreERt2* (Hasson et al., 2007) transgene have been described previously. *Prx1Cre(98)* is an independent transgenic line generated with the same construct used to produce the *Prx1Cre* (Logan et al., 2002) transgenic. Cre activity in the limbs is detected at slightly later stages (E8.5-E9.0) than that reported for the orginal *Prx1Cre* line.

### Tamoxifen induction

TM preparation and induction was done as described in the Joyner lab webpage (HYPERLINK “http://saturn.med.nyu.edu/research/dg/joynerlab/protocols.html” http://saturn.med.nyu.edu/research/dg/joynerlab/protocols.html). Adult dam females were gavaged with 6.5mg of TM in corn oil or by intra-peritoneal injection of 6mg 4-hydroxy-tamoxifen in 1:10 (v/v) ethanol: sunflower oil (from a stock of 20mg/ml) at the indicated time points. Cre activity from deleter strains, including *RosaCreERt2* used here, has been reported to cause apoptosis in embryos (Naiche and Papaioannou, 2007a). Muscle and tendon patterning phenotypes were only observed in animals homozygous for *Tbx5*, *Tbx4* or β-*Catenin* conditional alleles and carrying a Cre deleter transgene. Animals heterozygous for either conditional allele and carrying a cre deleter transgene had entirely normal limb muscle and tendon pattern and in most cases these are the control examples shown.

### Quantitative PCR

RNA was extracted from E12.5 *Tbx5*-deleted or heterozygous limbs using the RNeasy Mini Kit (Qiagen) and cDNA was subsequently prepared using SuperScript III Reverse Transcriptase (Invitrogen). 50 ng cDNA were loaded per qPCR well. The following TaqMan probes (Applied Biosystems) were used: *GAPDH* (4352932), *Cdh2* (682189), *β-Catenin* (705555), *Tcf4* (676819).

### In situ hybridization

Whole-mount and section in situ hybridisation was carried out essentially as previously described (Riddle et al., 1993; Schaeren-Wiemers and Gerfin-Moser, 1993). A minimum of three mutant embryos were analyzed at each stage described with each probe. Most probes have been described previously: *MyoD* (Davis et al., 1987), *SDF1α/β* (Vasyutina et al., 2005), *Osr1/2* (Stricker et al., 2006), *TCF4* (Kardon et al., 2003), *Mox2* (Mankoo et al., 1999), *SF/HGF* (kindly provided by F. Maina), *β-Catenin* (Hill et al., 2005), *N-Cadherin* (kindly provided by M. Takeichi).

### Immunohistochemistry

Whole mount immuno-histochemistry and OPT analysis were done as previously described (DeLaurier et al., 2006). Antibodies used – mouse anti-skeletal myosin (My32; 1:800; Sigma), mouse anti-Tcf4 (6H5-3; 1:100; Upstate), rabbit anti-N-Cadherin (abcam; 1:500), mouse anti-N-Cadherin (GC4; 1:200; Sigma), mouse anti-MyoD1 (1:50; Dako), mouse anti-β-Catenin (1:200; Sigma), rabbit anti-β-Catenin (1:500; Sigma); mouse anti-sarcomeric myosin (MF20; 1:20; DSHB); rabbit anti-Cadherin 11 (1:800; kindly given by R. Mege (Marthiens et al., 2002)).

### Histology

For histology, limbs were fixed in 4% PFA, dehydrated in a graded series of ethanol, cleared in xylene and embedded in fibrowax (VWR International, UK). Sections of 6-μm thickness were stained with hematoxylin and eosin (H&E).

## Supplementary Material

1

2

3

4

5

6

7

8
