# Supplementary material for: Tbx4 and Tbx5 acting in connective tissue are required for limb muscle and tendon patterning
Source: Dev Cell. Author manuscript; Available in PMC 2011 Feb 7. (PMC3034643; doi:10.1016/j.devcel.2009.11.013)
Supplement: 1 [file NIHMS164560-supplement-1.doc]

**Supplementary material**

Table S1

Summary of temporal deletions and resulting musculoskeletal abnormalities in forelimbs and hindlimbs.

MovieS1

OPT movie of wholemount My32 staining of E15.5 control limb shown in Fig. 1c (*Tbx5^lox/+^; Prx1CreERt2*).

MovieS2

OPT movie of wholemount My32 staining of E15.5 Tbx5 mutant limb shown in Fig. 1d (*Tbx5^lox/lox^; Prx1CreERt2*).

Figure S1

Misexpression of a dominant-negative form of Tbx5 in the chick wing causes disruption of muscle pattern.

Blocking Tbx5 activity by injecting a RCAS-Tbx5^EN^ virus at Hamburger Hamilton (HH) st. 16 leads to similar muscle patterning defects observed in *Prx1CreERt2; Tbx5^lox/lox^* mice as detected using the MF20 antibody at HH st. 36-37 and compared to uninjected controls (A,C); i.e. lack of muscles (cf. A,B) or ectopic muscle splitting and assuming a defective final size (cf. C,D).

Figure S2

#### Early disruption of muscle and tendon pattern following the deletion of Tbx4

Whole mount in situ hybridization shows aberrant muscle pattern marked by *MyoD,* observed at E12.5 (A,B) and at E13.5 (C,D) following the deletion of *Tbx4* at E9.5. Similarly, tendon pattern marked by *Scx* expression is disrupted in the *Tbx4*-deleted limbs (E,F). Control (*wild-type*) littermates (A,C,E) show normal muscle and tendon pattern.

Figure S3

*Tbx5 acts non-autonomously to pattern limb muscles*.

Whole mount immunohistochemistry demonstrates that muscle patterning in limbs in which *Tbx5* was deleted in the myoblasts prior to their migration into the limb field using the *Pax3CreKI* deleter line is not affected and resembles that of the control (*wild-type*) littermates (A,C) indicating that *Tbx5* acts in a non-autonomous manner in regulating limb muscle pattern. (A,C dorsal views; B,D ventral views).

Figure S4

*Loss of Tbx4 reduces β-Catenin and N-Cadherin in muscle connective tissue.* Immunohistochemistry of transverse sections of hindlimbs at E12.5 shows that deletion of *Tbx4* by administering TM at E9.5 leads to a specific loss of β-Catenin (green) in the MCT cells expressing Tcf4 (red) (A,B). Likewise N-Cadherin expression is reduced in these Tcf4 expressing MCT cells (C,D). Tagged panels show high magnifications of boxed areas (A-D). White arrows mark Tcf4 positive connective tissue domain whereas red arrows mark Tcf4 negative domains.
