# Supplementary material for: Tbx4 and Tbx5 acting in connective tissue are required for limb muscle and tendon patterning
Source: Dev Cell. Author manuscript; Available in PMC 2011 Feb 7. (PMC3034643; doi:10.1016/j.devcel.2009.11.013)
Supplement: 2 [file NIHMS164560-supplement-2.doc]

Table S1: Summary of temporal deletions and resulting musculoskeletal abnormalities

| *Forelimb – Tbx5 deletion* | *TM administration time* | *Skeletal abnormalities* | *Muscle and tendon abnormalities* |
| --- | --- | --- | --- |
|  | E8.5 | Yes (triphalangeal thumb, supernumerary thumb, hole in scapula, lack of deltoid tuberosity; Hasson et al., 2007) | Yes (muscles and tendons throughout the limb are affected) |
|  | E9.5 | Yes (triphalangeal thumb, supernumerary thumb, lack of deltoid tuberosity; Hasson et al., 2007) | Yes (muscles and tendons throughout the limb are affected) |
|  | ***E10.5*** | ***No*** (Hasson et al., 2007) | ***Yes*** *(muscles and tendons throughout the limb are affected)* |
|  | E11.5 | No | No |

| *Hindlimb – Tbx4 deletion* | E8.5 | Yes (Naiche and Papaioannou, 2007) | Yes (muscles and tendons throughout the limb are affected) |
| --- | --- | --- | --- |
|  | E9.5 | Yes (Naiche and Papaioannou, 2007) | Yes (muscles and tendons throughout the limb are affected) |
|  | E10.5 | Yes (Naiche and Papaioannou, 2007) | Yes (muscles and tendons affected are located mainly, *but not strictly*, in the foot plate) |
|  | ***E11.5*** | ***No*** | ***Yes*** (muscles and tendons affected are located *mainly* in the foot plate) |
|  | E12.5 | No | No |
