# Supplementary figures and images for: Tbx4 and Tbx5 acting in connective tissue are required for limb muscle and tendon patterning
Source: Dev Cell. Author manuscript; Available in PMC 2011 Feb 7. (PMC3034643; doi:10.1016/j.devcel.2009.11.013)

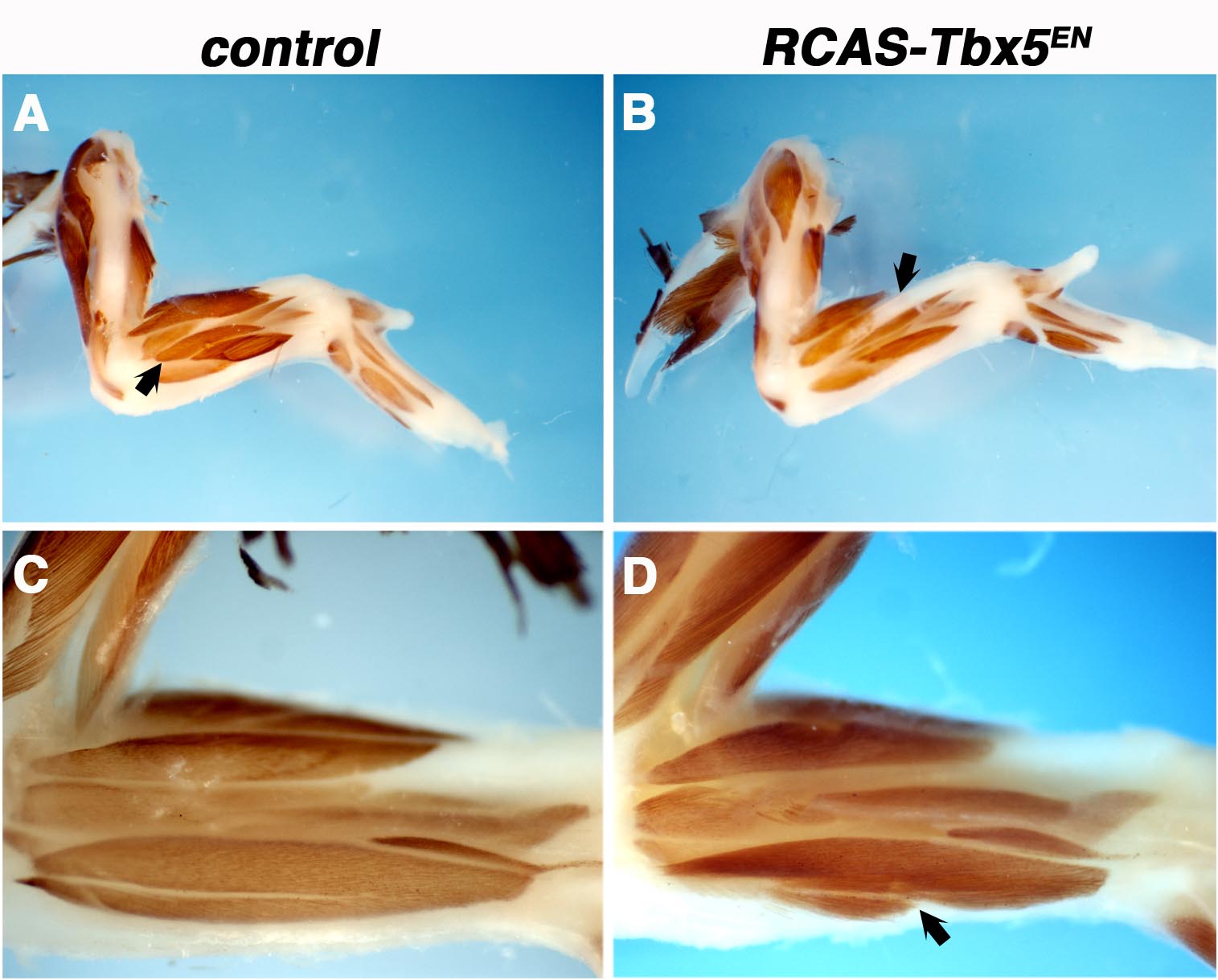

Supplement: 3 [file NIHMS164560-supplement-3.jpg]

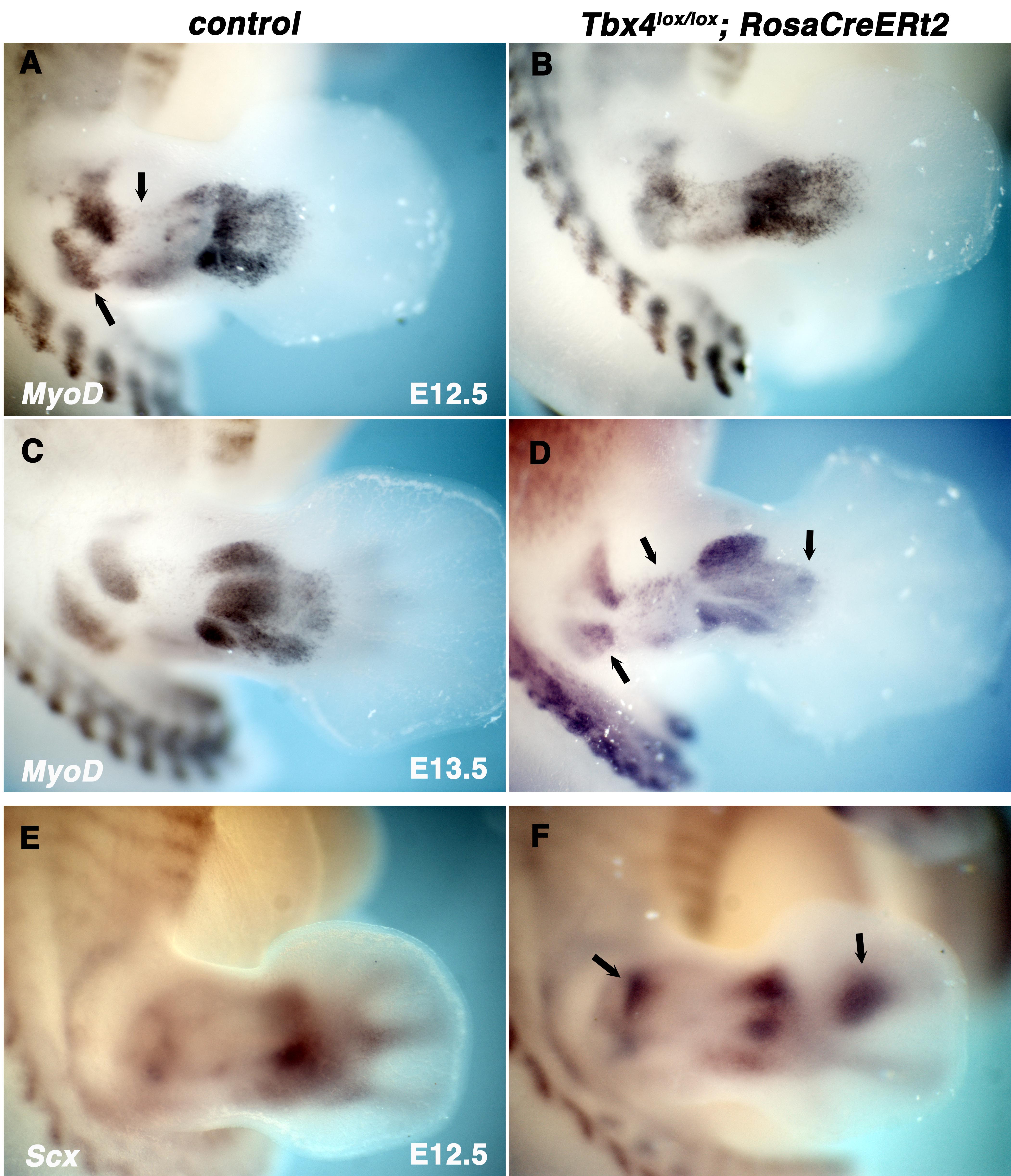

Supplement: 4 [file NIHMS164560-supplement-4.jpg]

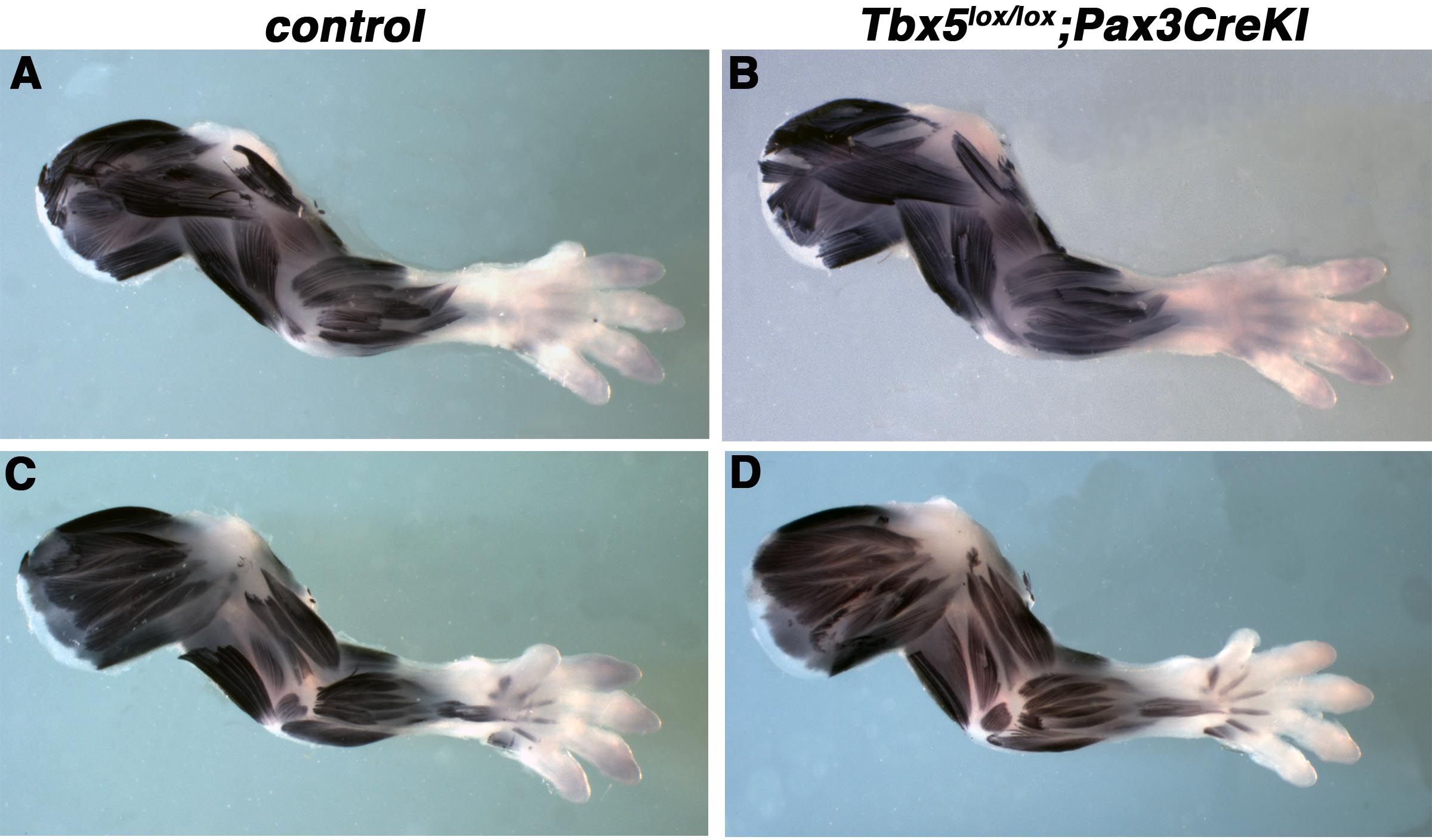

Supplement: 5 [file NIHMS164560-supplement-5.jpg]

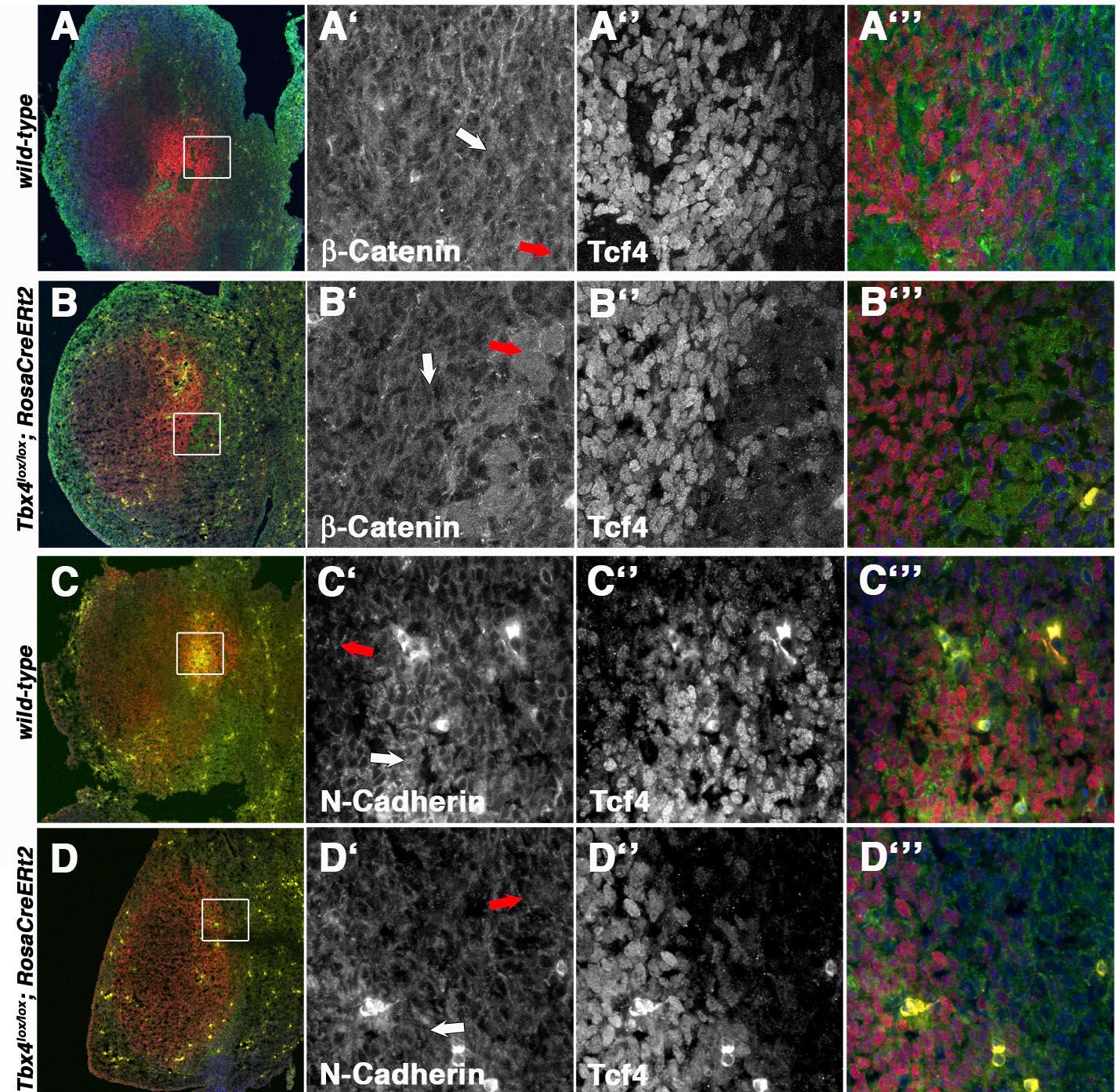

Supplement: 6 [file NIHMS164560-supplement-6.jpg]
